# A novel *MERTK* deletion is a common founder mutation in the Faroe Islands and is responsible for a high proportion of retinitis pigmentosa cases

**Published:** 2011-06-04

**Authors:** Elsebet Ostergaard, Morten Duno, Mustafa Batbayli, Kaj Vilhelmsen, Thomas Rosenberg

**Affiliations:** 1 Department of Clinical Genetics 4062, Copenhagen University Hospital Rigshospitalet, Copenhagen, Denmark; 2 Department of Ophthalmology, National Hospital of the Faroe Islands, Tórshavn, Faroe Islands; 3 Gordon Norrie Center for Genetic Eye Diseases, National Eye Clinic, Kennedy Center, Glostrup, Denmark

## Abstract

**Purpose:**

The aim of the study was to elucidate the genetic background of retinitis pigmentosa (RP) in a Faroe Islands population, a genetic isolate in the North Atlantic Ocean.

**Methods:**

Blood samples were collected from subjects diagnosed with RP and their families. DNA from affected individuals underwent single nucleotide polymorphism microarray analysis and homozygosity mapping followed by sequence analysis of candidate genes.

**Results:**

We identified 25 cases of nonsyndromic RP corresponding to a prevalence of 1 in 1,900. Single nucleotide polymorphism analysis revealed a homozygous region on chromosome 2q, common to patients in four families, which harbored the RP gene MER tyrosine kinase protooncogene (*MERTK*). A deletion of 91 kb was identified in seven patients, representing 30% of the analyzed Faroese cases of nonsyndromic RP. The clinical course of six patients who were homozygous for the deletion showed onset in the first decade followed by a rapid deterioration of both rod and cone photoreceptor function. Early macular involvement was present, in accordance with that of other reported patients with *MERTK* mutations.

**Conclusions:**

Previous studies have shown a frequency of less than 1% of *MERTK* mutations in RP patients. The 91-kb deletion encompassing exons 1–7 of *MERTK* is a common founder mutation in the Faroe Islands, responsible for around 30% of RP, and together with mutations in protocadherin 21 (*PCDH21*) accounts for more than half of the retinal dystrophy cases.

## Introduction

The Faroe Islands are a self-governing region of Denmark and consist of 18 small islands situated in the North Atlantic Ocean, 300 km northwest of Scotland between Iceland and Norway. The islands were inhabited by Norse Vikings about 1,000 years ago, and over the centuries the population remained small and isolated. This isolation combined with a recent expansion in population size to around 48,000 inhabitants explains a high incidence of certain autosomal-recessive disorders as a result of founder effects [1-3]. We identified retinitis pigmentosa (RP; OMIM 268000) as an additional disorder with a high prevalence in the Faroe Islands.

RP is characterized by progressive visual loss due to abnormalities of retinal photoreceptors. In the early stages, rod function is predominantly impaired, leading to defective dark adaptation and night blindness. This is followed by impairment of visual acuity due to loss of cone cells and eventually complete blindness.

The reported prevalence of RP varies from 1 in 4,000 to 1 in 2,500 [4]. Autosomal-recessive inheritance is found in 15%–25% of RP, autosomal-dominant in 5%–20%, X-linked-recessive in 5%–15%, and simplex (unknown inheritance) in 40%–50% [5]. A large number of genes responsible for autosomal-recessive RP have been identified, each responsible for only a small percentage of RP. One of these genes is mer tyrosine kinase protooncogene (*MERTK*; GenBank NM_006343). Shortly after the identification of a *Mertk* mutation in a rat—an extensively studied model of retinal dystrophy [6]—at the Royal College of Surgeons (RCS), Gal and coworkers reported the first three mutations in the human ortholog *MERTK* [7]. To date 11 pathogenic sequence variants have been published in seven unrelated individuals or families [8-14]. Three of the mutations were detected in consanguineous families originating in the Middle East [10], Saudi Arabia [13], Spain [11], and Morocco [12]. Screening of DNA samples from individuals with autosomal-recessive RP (ARRP) showed that *MERTK* mutations are a rare cause of retinal dystrophy in humans, affecting less than 1% of the probands [7,9,10,12,13].

## Methods

### Human subjects

Probands with RP, where one or both parents were born in the Faroe Islands, were identified from the files at the National Eye Clinic, Hellerup, and the Eye Department, National Hospital of the Faroe Islands, Tórshavn, Faroe Islands. Clinical data were obtained retrospectively from patient files from 1946 through 2004. Apart from RP, the patients were healthy and had no other eye disorders. Blood sampling from probands and family members was performed in 2007, according to an agreement with the Genetic Biobank, which also supplied genealogical information. Peripheral blood samples were collected in EDTA vacutainer tubes and sent to the Genetic Biobank, where DNA extraction was performed using the QIAamp DNA Mini Kit (Qiagen, Dusseldorf, Germany). The study adhered to the tenets of the Helsinki Declaration and was approved by the Faroese Scientific Ethical Committee. Consent was obtained from all participants after oral and written information was provided.

### Microarray analysis and homozygosity mapping

DNA from 19 patients with RP was used for a genome-wide search for homozygosity with the Affymetrix GeneChip 50K Xba array (Affymetrix Inc., Santa Clara, CA). In brief, 250 ng of DNA was digested with the restriction enzyme XbaI, mixed with Xba adapters, and ligated with the T4 DNA ligase. The ligated DNA was PCR-amplified in four PCR reactions, pooled, and purified. The purified PCR product was fragmented with DNase I and end-labeled with biotin. The samples were hybridized to an array for 18 h in a hybridization oven. The array was washed, stained, and scanned with an Affymetrix GeneChip scanner 3000. Affymetrix software was used to analyze the data that was exported to an Excel file and manually inspected for homozygous regions.

### Mutation analysis

PCR was performed using the Promega (Madison, WI) GoTaq system with the following conditions: 0.2 mM deoxyribonucleotide triphosphate (dNTPs), 1× buffer, 1.5 mM MgCl_2_, 0.5 mM of each forward and reverse primer (Table 1), 10 ng template, and 0.5 U polymerase in a total volume of 50 µl. The PCR program was: 94 °C for 2 min, 35 cycles of denaturing at 94 °C for 30 s, annealing at 60 °C for 30 s, and extension at 72 °C for 30 s, and a final extension step of 72 °C for 7 min.

**Table 1:** Primer sequences for amplification of *MERTK*.

**Primer sequence (5′-3′)**	**Exon**	**Primer sequence (5′-3′)**	**Exon**
CCACTCGGCACTCACTG	1	GAAACACGTTTCTTCTAGGGG	11
AGTGGGTGGAGGGTGTTT		TGCTGCTTTTAAAGACATTTTG	
TGTGTGTGTTAATGAATTCTGCT	2	AAGGTTGCACCATTGCAC	12
GAACTCAGGAGGTGGAGG		GTGCCAGATCTGAGTTTCAA	
TGCGTACAATGGCCTAGC	3	TGGTTTGCGATGTGGG	13
TGCTGATAGATTTGCAAGGTT		CTTGGTGACCAGTGTTCCA	
GAGCAAGACTCCATCCCC	4	AGCCCTACTAGCCCCTGA	14
CCCATAACTTTGCTGCCA		CTGGGTGAAAACCTCAATGT	
TCTCCATAGCTAGCACACCTTT	5	TGTGAGTTAGGCGCTCTTG	15
TGAGTTGTCCAAGGTCACAA		CAACCCTGGACACACAGG	
GGGAGGCTCCTCTTGAAA	6	TTGCCAAGAAGTTTAAGGTTT	16
GCTGGACACTGAAGCAGG		AAGCCACACCATCCCC	
CCCAGAAAGGAAGCAGGT	7	CACAGGCTGGTGGTGTCT	17
CGCCTCAATAATTTTCTCCTC		TCTGAGCAAGCTGCCAAC	
TTGAAAAGGTGAAAATGTGCT	8	AGCAGTGCGTCTCACACA	18
TGAAAGGTGCCTTGCCTA		GTGTCTGTGGGTTCCACC	
ATGCTGTGGAAGTGTGGC	9	TTGTATAAATATTAGGCCACCAAA	19A
ACTCCTCCTGCTTTTGCC		CCCATTCAGGATGTACCG	
TCGCATGGTCTCAGCTTAC	10	CCACTGGACTTGAACATCG	19B
GGGTATGCATAAGGCAGG		TTCATCTGGTGCTTTGGG	

Sequencing was performed with the ABI Big Dye Terminator version 1.1 Cycle Sequencing Kit (Applied Biosystems, Foster City, CA). The sequencing reactions were vacuum purified with the Montage Seq96 Sequencing Reaction Cleanup Kit (Millipore SA, SaintQuentin, France) and analyzed on an ABI 3130xl Gene Analyzer (Applied Biosystems).

The data were analyzed with Sequencing Analysis version 5.2 software (Applied Biosystems), and mutation screening was performed using Mutation Surveyor version 3.1 software (SoftGenetics, State College, PA).

To screen for the 91-kb deletion, a triple-primer PCR assay was designed: the forward primer (5′-GTC ATT ATT GAG CTC AGT GCG-3′) was located immediately upstream of the deletion junction (5′-AAA TTG AAA ATA ATA TGC CAG GC-3′), whereas one reverse primer spanned the deletion junction, resulting in a deletion-specific PCR product of 264 bp; the other reverse primer (5′-CTG GCT CTT GTC ATT TCC TG-3′) amplified the wild-type allele, which was seen as a 384-bp product. Primer sequences and PCR conditions are available on request.

## Results

### Clinical findings

Forty-three individuals with panretinal dystrophies were identified in the population of 48,000, corresponding to a prevalence of 1 in 1,100. Of these, 17 out of 18 syndromic cases were diagnosed with Bardet–Biedl syndrome. The remaining 25 cases of nonsyndromic RP were either isolated cases or belonged to one of three families (882, 232, 801) with three or more affected family members. The prevalence of nonsyndromic RP in the Faroe Islands is thus 1 in 1,900.

Twenty-one individuals with nonsyndromic RP consented to participate in the study. Six of these belonged to a family with a distinct phenotype in which a mutation in protocadherin 21 (*PCHD21*) has been identified [15].

**Figure 1:**
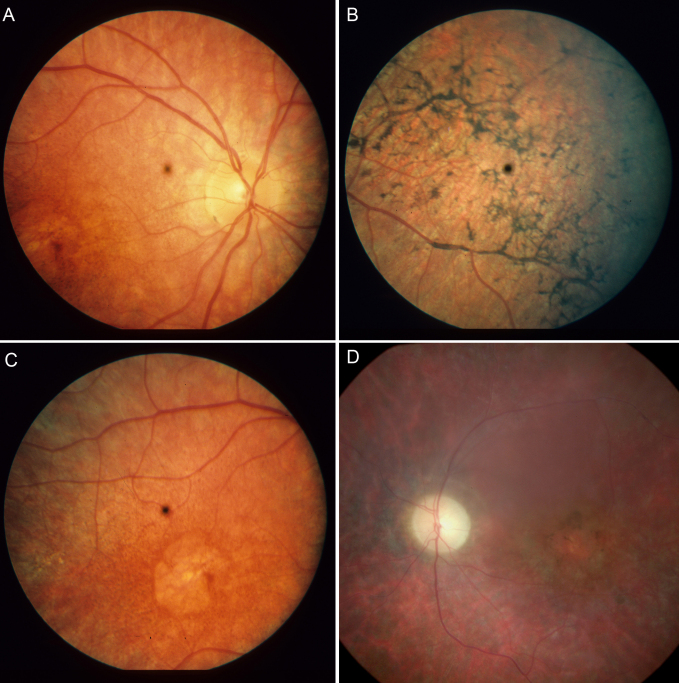
Fundus aspects from two patients homozygous for a MER tyrosine kinase protooncogene (*MERTK*) deletion. **A**-**C**: 22-year-old female (patient 106); the central black spot. The central black spot is an artifact to reduce reflexes from the Zeiss fundus camera. **A**: In the posterior part of the retina, near-normal calibrated central arterioles and a normal-appearing optical nerve head are evident. **B**: In the inferotemporal aspect, widespread mottled pigment epithelial atrophy and heavy pigment aggregates partly sheeting the retinal venules are seen. **C**: In the macular region, distinct, nearly circular, foveal atrophy is present. **D**: In the left eye from a female age 35 years (patient 232), marked atrophy of the optic papilla, constricted arterioles, and distinct central pigment atrophy are present.

The clinical information that was available covered a time span of 60 years and included many different examination procedures (Table 2). No new examinations were performed. The patients with *MERTK* mutations had a severe phenotype with onset of night blindness in the first decade, a rapidly progressive deterioration of central vision, and severely affected visual fields during the second decade. Full-field ERGs were mostly nondetectable at first examination, but in the youngest individuals residual rod and cone responses were still recordable. Dark adaptation showed some residual cone activity but no signs of rod activity. Blindness was mostly complete or near complete at the age of 40. Fundus pictures (Figure 1) revealed a generalized, diffuse, retinal pigment epithelial (RPE) atrophy with early mottling, followed by more or less pronounced peripheral hyperpigmentation and marked central atrophy. Narrow vessels and pale optic discs appeared relatively late but with large interindividual variation.

**Table 2:** Clinical findings in six homozygous and one heterozygous individuals carrying the Faroese *MERK* founder mutation.

**ID**	**Age of onset**	**Age at first/last exam**	**Visual acuity at first exam**	**Visual acuity at last exam**	**Refraction right**	**Refraction left**	**Full-field ERG (ffERG)**	**Perimetry (age)**	**Dark adaptation**
186	9	16/32	6/12 6/12	1/60 1/60	+2.50–1.50x10°	+2.50–0.50x170°	Flat	TV 2–3° (32)	No rod activity
232	8	9/35	6/7.5 6/9	6/60 0.5/60	−4.50–3.00x0°	−4.50–3.00x0°	Flat	Paracentral islands (35)	No rod activity
113	6	24/32	6/36 6/24	HM 0.5/24	Emmetropia	Emmetropia	N.d.	TV 1–2°	N.d.
106	5	6/30	6/6 6/9	1/69 1/60	+0.50	0.00–1.25x160°	Sub normal	TV 3–5°	N.d.
102	10	24/27	2/60 3/60	1/60 1/60	Emmetropia	Emmetropia	N.d.	TV 5° plus peripheral islands	N.d.
249*	8	6/35	6/6 6/6	6/12 6/18	−6.00–0.50x90°	−6.50	Reduced‡	20–50°	Diphasic. Threshold elevation 1 log unit

Abbreviations N.d.: not done, HM: hand movements, TV: tunnel vision, *: heterozygous.

### Genetic analyses

**Figure 2:**
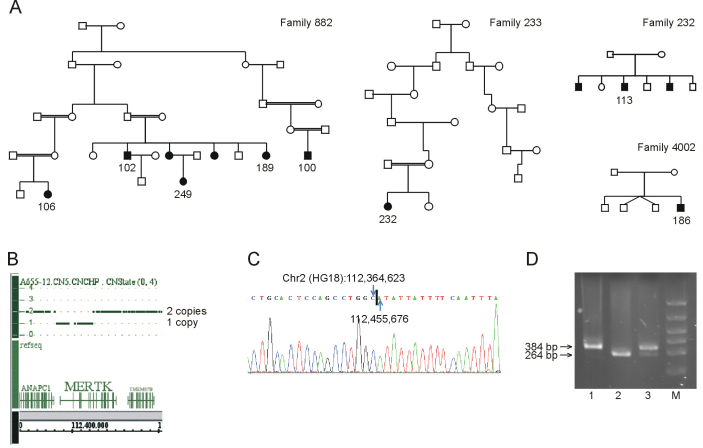
Pedigrees and analysis of the MER tyrosine kinase protooncogene (*MERTK*) deletion. **A**: Pedigrees of four Faroese families (882, 232, 233, 4002) with the *MERTK* deletion. **B**: Single nucleotide polymorphism (SNP) copy number analysis of a heterozygous carrier showing that the deletion has a size of 91 kb. **C**: Sequence showing the breakpoints of the 91-kb deletion in a homozygous patient. **D**: The triple-primer PCR assay generating a deletion-specific fragment of 264 bp and a wild-type fragment of 384 bp; lane 1, wild-type; lane 2, homozygous patient; lane 3, heterozygous carrier; lane M, molecular marker.

A genome-wide search for homozygosity revealed a homozygous region on chromosome 2q that was common to patients from four families (Figure 2A). The core haplotype was 6.1 Mb (positions 111.6–117.7 Mb) and included the RP gene *MERTK* (Appendix 1). Sequence analysis of exons 8–19 in *MERTK* in patient 186 was normal, whereas exons 1–7 could not be amplified by PCR. Single nucleotide polymorphism (SNP) analysis in an obligate heterozygous carrier showed a deletion of an estimated 91 kb encompassing exons 1–7 (Figure 2B). PCR across the estimated deletion resulted in an abnormal PCR product harboring the deletion breakpoints. Subsequent sequencing identified the exact breakpoint of the deletion, which can be described as chr2 (HG18):112,364,622_112,455,675del91,054 (Figure 2C). A targeted PCR assay was developed allowing carrier screening for the deletion. This analysis showed that the seven patients, from whom DNA was available, were homozygous for the deletion, whereas seven family members were carriers (Figure 2D).

Patient 249 from family 882 had a father unrelated to the other family members. She was heterozygous for the deletion, but sequencing of *MERTK* did not reveal another mutation, indicating that RP could be caused by mutations in a different gene in this patient.

Screening of 94 anonymous Faroese controls revealed three heterozygous carriers, and thus the carrier frequency is approximately 3%. Assuming Hardy–Weinberg equilibrium, the carrier frequency corresponds to an incidence of RP caused by *MERTK* mutations in the Faroe Islands of around 1 in 3,940. This is in concordance with the observed prevalence of 11 affected patients in a population of 48,000, i.e., 1 in 4,360.

## Discussion

The high prevalence of nonsyndromic RP of 1 in 1,900 in the Faroe Islands can partly be explained by a founder effect, with a *MERTK* deletion being responsible for around 30% of RP and a *PCDH21* mutation responsible for around 25% [15]. Linkage data suggest that the remaining RP cases are caused by mutations in several other genes (data not shown).

The clinical picture of the patients with a *MERTK* deletion reported here is very similar to descriptions in previous reports showing a severe rod–cone dystrophy with early onset in the first decade (summarized in [13]). In addition to symptoms seen in other generalized retinal dystrophies, the fundi of patients with *MERTK* mutations develop an early bull’s eye central atrophy, which may guide the mutation analysis. In contrast to early onset retinal dystrophies of Leber type that mostly exhibit high hypermetropia, emmetropia or low myopia was present in most Faroese *MERTK* patients.

The heterozygous patient 249 from family 882 (Figure 2A) with apparent pseudodominant inheritance of RP was 8 years old when examined the first time. She is now 54 years old and nearly blind. Her fundi are typical for RP with narrow arterioles, pale optic nerves, and peripheral spicule formation. The diagnosis of RP is undoubtedly correct, but her symptoms are milder than in the homozygous patients. A second *MERTK* mutation was not identified, leaving the possibility that her RP is caused by mutation(s) in a different gene.

In the RCS rat and mouse, which are naturally occurring *MERTK* knockout models, retinal photoreceptor dystrophy is a consequence of a defect in phagocytosis of shed outer segments by the retinal pigment epithelium. This results in accumulation of outer segment debris in the subretinal space; the retinal pigment epithelium and the photoreceptors lose contact and the retina degenerates. Spectral domain optical coherence tomography in humans with *MERTK* mutations has shown thinning of the outer retinal layers and a debris-like material in the sub-neurosensory space, which is similar to findings in the knockout models [12,13], indicating an analogous disease mechanism in humans.

Subretinal *Mertk* gene transfer in young RCS rats has shown photoreceptor survival due to a correction of phagocytotic activity by RPE cells, resulting in preservation of retinal function [16-18]. Patients with Leber congenital amaurosis caused by *RPE65* mutations have been treated successfully by gene replacement therapy [19-21]. *RPE65* deficiency causes visual impairment due to depletion of visual pigment from the phototransduction cascade, but the anatomic features of the retina are well preserved. In addition, RPE65 is specific to the retinal pigment epithelium, and subretinal injection of viral shuttles can more efficiently transduce retinal pigment epithelium than the photoreceptor cells, possibly because of anatomic barriers. *MERTK*-related retinal dystrophies share many features with those due to *RPE65* mutations and could therefore be a good target for future viral-mediated gene therapy in humans.

## Appendix 1.

Haplotypes of seven patients with retinitis pigmentosa of the region on chromosome 2 surrounding *MERTK.* To access the table, click or select the words “Appendix 1.” This will initiate the download of a pdf archive that contains the table.
